# Supplementary material for: Identification of a Novel Equine Papillomavirus in Semen from a Thoroughbred Stallion with a Penile Lesion
Source: Viruses. 2019 Aug 4;11(8):713. doi: 10.3390/v11080713 (PMC6723834; doi:10.3390/v11080713)
Supplement: Supplementary file 1 [file viruses-11-00713-s001.zip › Li.Table S3.docx]

Table S3. Genomic nucleotide and amino acid features of the genus *Dyoiotapapillomavirus*

|  | ***Dyoiotapapillomavirus 1*** | | ***Dyoiotapapillomavirus 2*** | |
| --- | --- | --- | --- | --- |
| **Accession number** | **HM461973** | **MN117918** | **NC_020085** | **NC_020084** |
|  | **EcPV2** | **EcPV9** | **EcPV4** | **EcPV5** |
| **Predicted nucleotide features*** |  |  |  |  |
| Genome size (bp) | 7803 | 7656 | 7554 | 7519 |
| GC content (%) | 56.0 | 52.9 | 54.9 | 50.2 |
| E2 binding site  ACC-N5-7-GGT | ACC-N4-GGT (2):1387,1991  ACC-N5-GGT (2): 587,5458  ACC-N6-GGT (2): 418,5823  ACC-N7-GGT (1): 948 | ACC-N4-GGT (2): 4564,6422  ACC-N5-GGT (4): 340,5142,5761,7032  ACC-N6-GGT (8): 78,137,255,313,355,476,549,4812  ACC-N7-GGT (2): 5781,7399 | ACC-N4-GGT (2): 4257,5033  ACC-N5-GGT (1): 3663  ACC-N6-GGT (3): 223,1455,2102,  ACC-N7-GGT (0) | ACC-N4-GGT (1):92  ACC-N5-GGT (2): 610,5059  ACC-N6-GGT (3): 87,222,266,  ACC-N7-GGT (1): 6240 |
| E1 binding site  A(A/T)GATTGTTGTTAACAAT | 771  AAGATTGTTGTGGACCAC | 580  TAGATCATTGTTAACAAC | 561 AAGATTGTTATTAGGAAT | 566  ATGATTGTGGTTAACAAC |
| Polyadenylation sites  AATAAA | 43 | 34 | 34, 4581,6706 | 44 |
| Sp1 binding sites  GGCGGG | 615 (LCR); 5337,5559, 5810, 7353 | 5021,5084 | 635 (LCR); 1721, 2706, 5593, 5996, 6812 | 398 (LCR); 1805, 3945, 4170 |
| NF1 binding sites  CGGAA | 3536, 3934, 4051,4205, 4525, 6065 | 2247,3144,5728, | 196, 893,1002, 2183, 3821 | 2037, 3834, 4299, 4853, 6315, 7046 |
| AP1 binding sites  TGANTCA | 727 | - | - | 523 |
| **Predicted amino acid features** |  |  |  |  |
| ATP-dependent helicase motive in E1 | GPPNTGKS 1363 (455) | GEPDTGKS  1339 (447) | GPPNTGKT 1357 (453) | GPPDTGKS 1372 (458) |
| Cyclin interaxion RXL motive in E1, KRRLF | 331 (111) | 325 (109) | 313 (105) | 334 (112) |
| Metal-binding motifs in E6  (CXXCX29CXXC) | 22 (8)-133 (44) 247 (83)-357 (118) | 28 (10)-138 (46)  253 (85)-364 (121) | 28 (10)-13 8(46) 250 (84)-36 1(120) | 28 (10)-138 (46) 250 (84)-361 (120) |
| Metal-binding motifs in E7  (CX2CX29CX2C) | 208 (70)-319 (106) | 148 (50)-259 (86) | 118 (40)-229 (76) | 116 (39)-226 (75) |
| Retinoblastoma tumour suppressor binding domain in E7 (LXCXE) | - | - | - | - |
| Nuclear localization signal in L2 | RKRKRR 1465 (489) | RKRKRR  1480 (494) | RRKRR 1426 (476) | RRKRR 1411 (471) |
| Nuclear localization signal in L1 | KKRK 1483 (495) | KKRR  1490 (497) | KRRKR 1471 (491) | KRRRK 1456 (486) |

* For all sequences nucleotide positions are numbered starting from the first nucleotide following the stop codon of the L1 ORFs.
