# Supplementary material for: Identification of a Novel Equine Papillomavirus in Semen from a Thoroughbred Stallion with a Penile Lesion
Source: Viruses. 2019 Aug 4;11(8):713. doi: 10.3390/v11080713 (PMC6723834; doi:10.3390/v11080713)
Supplement: Supplementary file 1 [file viruses-11-00713-s001.zip › Li.Table S2.docx]

Table S2. Information on the RNA sequencing libraries generated here.

| Libraries | Samples types | Collected Type | Paired End (100bp) | Data |
| --- | --- | --- | --- | --- |
| 3UR | Urine | Standard with RNAlater | 108,274,738 | 21.87 Gb |
| 4SR | Semen | Standard with RNAlater | 101,908,087 | 20.59 Gb |
| 6UP | Urine | Standard | 97,162,843 | 19.63 Gb |
| 6SP | Semen | Standard | 111,117,835 | 22.45 Gb |
| Total |  |  | 418,463,503 | 84.54 Gb |
