# Supplementary material for: Identification of a Novel Equine Papillomavirus in Semen from a Thoroughbred Stallion with a Penile Lesion
Source: Viruses. 2019 Aug 4;11(8):713. doi: 10.3390/v11080713 (PMC6723834; doi:10.3390/v11080713)
Supplement: Supplementary file 1 [file viruses-11-00713-s001.zip › Li.Figure S3.pdf]

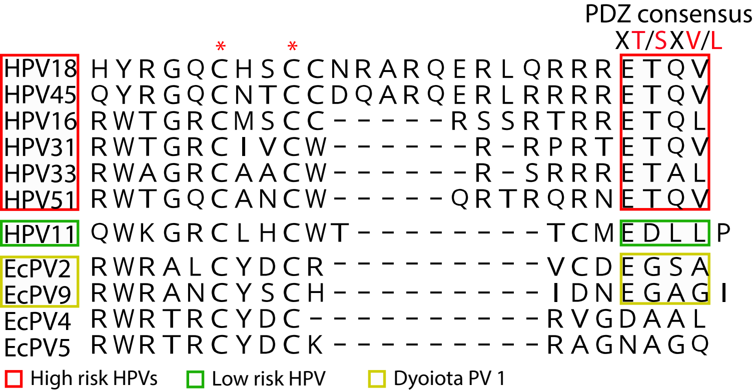

PDZ consensus
XT/SXV/L
*
*
HPV18 HYRGQCHSCCNRARQERLQRRRETQV
HPV45 QYRGQCNTCCDQARQERLRRRRETQV
HPV16 RWTGRCMSCC------RSSRTRRETQL
HPV31 RWTGRCIVCW-------R-RPRTETQV
HPV33 RWAGRCAACW-------R-SRRRETAL
HPV51 RWTGQCANCW------QRTRQRNETQV
HPV11 QWKGRCLHCWT---------TCMEDLLP
EcPV2 RWRALCYDCR----------VCDEGSA
EcPV9 RWRANCYSCH----------IDNEGAGI
EcPV4 RWRTRCYDC-----------RVGDAAL
EcPV5 RWRTRCYDCK----------RAGNAGQ
High risk HPVs
Low risk HPV
Dyoiota PV 1
