# Supplementary material for: Identification of a Novel Equine Papillomavirus in Semen from a Thoroughbred Stallion with a Penile Lesion
Source: Viruses. 2019 Aug 4;11(8):713. doi: 10.3390/v11080713 (PMC6723834; doi:10.3390/v11080713)
Supplement: Supplementary file 1 [file viruses-11-00713-s001.zip › Li.Figure S2.pdf]

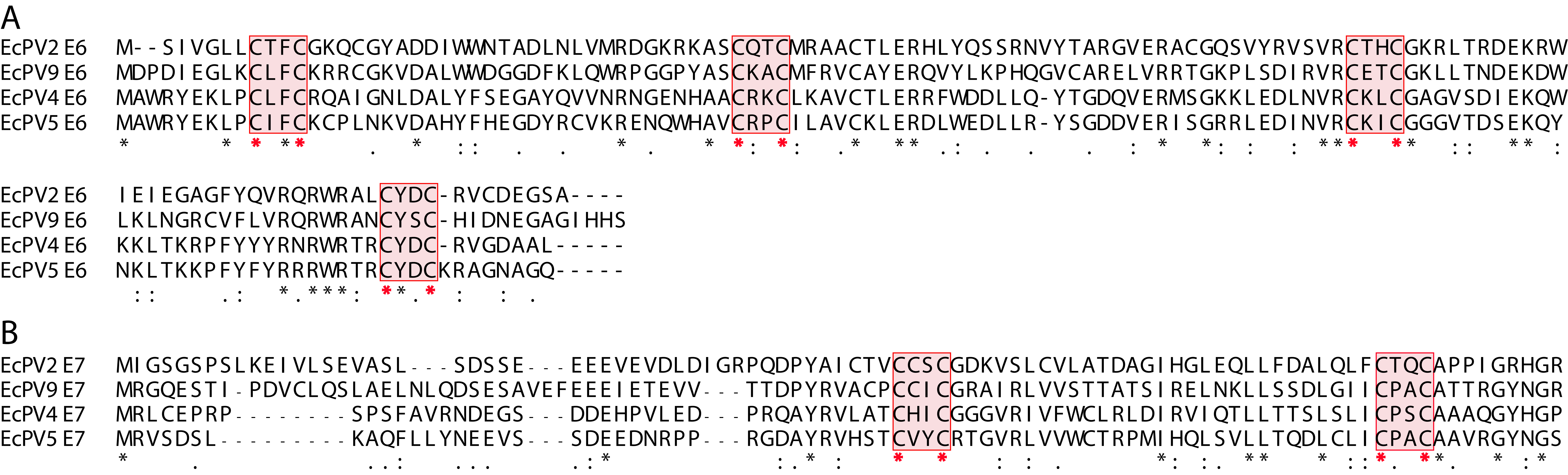
A
EcPV2 E6 M--SIVGLLCTFCGKQCGYADDIWWNTADLNLVMRDGKRKASCQTCMRAACTLERHLYQSSRNVYTARGVERACGQSVYRVSVRCTHCGKRLTRDEKRW
EcPV9 E6 MDPDIEGLKCLFCKRRCGKVDALWWDGGDFKLQWRPGGPYASCKACMFRVCAYERQVYLKPHQGVCARELVRRTGKPLSDIRVRCETCGKLLTNDEKDW
EcPV4 E6 MAWRYEKLPCLFCRQAIGNLDALYFSEGAYQVVNRNGENHAACRKCLKAVCTLERRFWDDLLQ-YTGDQVERMSGKKLEDLNVRCKLCGAGVSDIEKQW
EcPV5 E6 MAWRYEKLPCIFCKCPLNKVDAHYFHEGDYRCVKRENQWHAVCRPCILAVCKLERDLWEDLLR-YSGDDVERISGRRLEDINVRCKICGGGVTDSEKQY
EcPV2 E6 IEIEGAGFYQVRQRWRALCYDC-RVCDEGSA----
EcPV9 E6 LKLNGRCVFLVRQRWRANCYSC-HIDNEGAGIHHS
EcPV4 E6 KKLTKRPFYYYRNRWRTRCYDC-RVGDAAL-----
EcPV5 E6 NKLTKKPFYFYRRRWRTRCYDCKRAGNAGQ-----
B
EcPV2 E7 MIGSGSPSLKEIVLSEVASL---SDSSE---EEEVEVDLDIGRPQDPYAICTVCCSCGDKVSLCVLATDAGIHGLEQLLFDALQLFCTQCAPPIGRHGR
EcPV9 E7 MRGQESTI-PDVCLQSLAELNLQDSESAVEFEEEIETEVV---TTDPYRVACPCCICGRAIRLVVSTTATSIRELNKLLSSDLGIICPACATTRGYNGR
EcPV4 E7 MRLCEPRP--------SPSFAVRNDEGS---DDEHPVLED---PRQAYRVLATCHICGGGVRIVFWCLRLDIRVIQTLLTTSLSLICPSCAAAQGYHGP
EcPV5 E7 MRVSDSL----------KAQFLLYNEEVS---SDEEDNRPP---RGDAYRVHSTCVYCRTGVRLVVWCTRPMIHQLSVLLTQDLCLICPACAAVRGYNGS
