# Supplementary material for: Identification of a Novel Equine Papillomavirus in Semen from a Thoroughbred Stallion with a Penile Lesion
Source: Viruses. 2019 Aug 4;11(8):713. doi: 10.3390/v11080713 (PMC6723834; doi:10.3390/v11080713)
Supplement: Supplementary file 1 [file viruses-11-00713-s001.zip › Li.Table S1.docx]

Table S1. Equine associated papillomaviruses, and their associated disease/syndrome.

| Virus | Disease/Syndrome in equid | References |
| --- | --- | --- |
| *Bovine papillomavirus type 1* | Equine sarcoid | 1 |
| *Bovine papillomavirus type 2* | Equine sarcoid | 1 |
| *Bovine papillomavirus type 13* | Equine sarcoid | 2 |
| *Equine caballus papillomavirus 1* | Classical papillomatosis | 3 |
| *Equine caballus papillomavirus 2* | Genital papillomas/papillomatosis, SCC, Aural plaques, White genital plaques | 1, 3, 4,13 |
| *Equine caballus papillomavirus 3* | Aural plaques | 3,5 |
| *Equine caballus papillomavirus 4* | Aural plaques, White genital plaques | 3,6 |
| *Equine caballus papillomavirus 5* | Aural plaques | 3,6 |
| *Equine caballus papillomavirus 6* | Aural plaques | 3,6 |
| *Equine caballus papillomavirus 7* | Genital papillomas/papillomatosis | 3,6 |
| *Equine caballus papillomavirus 8* | Generalized papillomatosis, SCC | 7 |
| *Equine caballus papillomavirus 9* | Genital wart-like | This study |
| *Equus asinus papillomavirus 1* | Sun associated cutaneous lesions | 8 |
| *Equus asinus papillomavirus 2* | - | Unpublished |
