# Supplementary figures and images for: Identification of a Novel Equine Papillomavirus in Semen from a Thoroughbred Stallion with a Penile Lesion
Source: Viruses. 2019 Aug 4;11(8):713. doi: 10.3390/v11080713 (PMC6723834; doi:10.3390/v11080713)

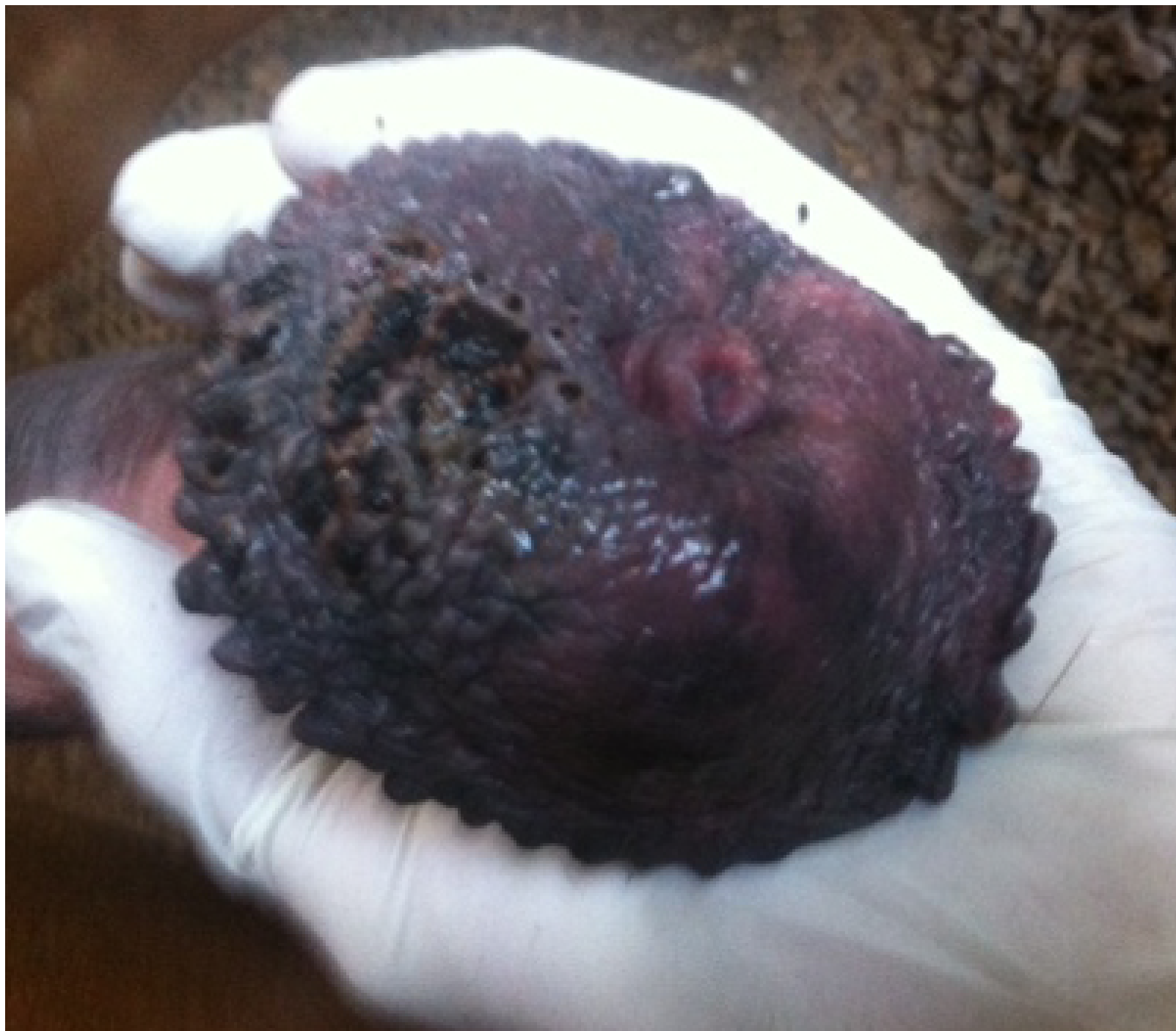

Supplement: Supplementary file 1 [file viruses-11-00713-s001.zip › Li.Figure S1.pdf]
